# Patterns and predictors of self‐reported clinical diagnosis and treatment for depression in prostate cancer survivors

**DOI:** 10.1002/cam4.2239

**Published:** 2019-05-20

**Authors:** Daniel O. Erim, Jeannette T. Bensen, James L. Mohler, Elizabeth T. H. Fontham, Lixin Song, Laura Farnan, Scott E. Delacroix, Edward S. Peters, Theodora N. Erim, Ronald C. Chen, Bradley N. Gaynes

**Affiliations:** ^1^ Department of Health Policy and Management, Gillings School of Global Public Health the University of North Carolina at Chapel Hill Chapel Hill North Carolina; ^2^ RTI International Research Triangle Park North Carolina; ^3^ Department of Epidemiology, Gillings School of Global Public Health University of North Carolina Chapel Hill North Carolina; ^4^ Lineberger Comprehensive Cancer Center, School of Medicine University of North Carolina Chapel Hill North Carolina; ^5^ Department of Urology, Roswell Park Comprehensive Cancer Center Buffalo New York; ^6^ Louisiana State University Health Sciences Center School of Public Health New Orleans Louisiana; ^7^ School of Nursing University of North Carolina at Chapel Hill Chapel Hill North Carolina; ^8^ Department of Urology Louisiana State University New Orleans Louisiana; ^9^ Department of Radiation Oncology University of North Carolina Chapel Hill North Carolina; ^10^ Department of Psychiatry University of North Carolina Chapel Hill North Carolina

**Keywords:** clinical diagnosis, clinical recognition, depression treatment, predictors, prostate cancer

## Abstract

**Background:**

Appropriate depression care is a cancer‐care priority. However, many cancer survivors live with undiagnosed and untreated depression. Prostate cancer survivors may be particularly vulnerable, but little is known about their access to depression care. The goal of this study was to describe patterns and predictors of clinical diagnosis and treatment of depression in prostate cancer survivors.

**Methods:**

Generalized estimating equations were used to evaluate indicators of self‐reported clinical diagnosis and treatment depression as a function of individual‐level characteristics within a longitudinal dataset. The data were from a population‐based cohort of North Carolinian prostate cancer survivors who were enrolled from 2004 to 2007 on the *North Carolina‐Louisiana Prostate Cancer Project* (N = 1,031), and prospectively followed annually from 2008 to 2011 on the *Health Care Access and Prostate Cancer Treatment in North Carolina* (N = 805).

**Results:**

The average rate of self‐reported clinical diagnosis of depression was 44% (95% CI: 39%‐49%), which declined from 60% to 40% between prostate cancer diagnosis and 5‐7 years later. Factors associated with lower odds of self‐reported clinical diagnosis of depression include African‐American race, employment, age at enrollment, low education, infrequent primary care visits, and living with a prostate cancer diagnosis for more than 2 years. The average rate of self‐reported depression treatment was 62% (95% CI: 55%‐69%). Factors associated with lower odds of self‐reported depression treatment included employment and living with a prostate cancer diagnosis for 2 or more years.

**Conclusion:**

Prostate cancer survivors experience barriers when in need of depression care.

## 1 INTRODUCTION

Major and persistent depressive disorders (or depression) affect one in five cancer survivors.^1^, ^2^ Adverse effects on the cost, quality, and duration of cancer survivorship justify prevention of depression as a cancer care priority.^3^, ^4^, ^5^, ^6^, ^7^, ^8^, ^9^, ^10^, ^11^ For example, since 2014, the American Society of Clinical Oncology recommends depression screening for all survivors at their initial visit, at appropriate intervals, and as clinically indicated, especially with changes in disease or treatment status (ie, posttreatment, recurrence, progression) and on transition to palliative and end‐of‐life care.^10^ Since 2015, the American College of Surgeons' Commission on Cancer required cancer centers in the United States to screen survivors for psychosocial distress.^11^ The 2016 US Preventive Services Task Force statement recommended depression screening and appropriate follow‐up care in all adults and emphasized that cancer survivors face higher risks for depression.^12^

Despite widespread support for depression care in cancer survivors, many depressed survivors live with undiagnosed or untreated depression due to low‐screening rates (4% in the general population),^11^, ^13^ poor clinician recognition,^14^, ^15^ and patients’ refusal/inability to get help.^16^, ^17^ Available evidence suggests that (a) 2‐3 in four depressed survivors do not receive a clinical diagnosis of depression; (b) 1‐2 in five survivors fail to initiate depression treatment despite having received a clinical diagnosis of depression; and (c) unequal access to depression care persists across sociodemographic groups.^18^, ^19^, ^20^, ^21^, ^22^, ^23^, ^24^ These gaps along the depression care pathway may impose dire consequences on the health and safety of cancer survivors.^25^, ^26^ Moreover, prostate cancer survivors may be the most vulnerable cancer survivor–group since men are usually reluctant to report depressive symptoms or seek mental health care.^27^, ^28^, ^29^ However, little is known about clinical diagnosis and treatment of depression in this patient population.

The overarching goal of this study was to describe rates, patterns and predictors of clinical diagnosis, and treatment of depression among prostate cancer survivors. These descriptions were based on self‐report, and the study was designed to: (a) provide insight into the reported racial difference in the risk of depression among prostate cancer survivors^30^; (b) describe gaps in depression care in the short‐ to medium‐term after prostate cancer diagnosis; (c) enhance the ability to plan and execute interventions that make depression care more accessible; and (d) respond to the request for “research to evaluate…the diagnostic process, and to identify, learn from and reduce diagnostic error… (including diagnosis that was unintentionally missed)” by the National Academy of Science, Engineering and Medicine's Committee on Diagnostic Error in Medicine.^31^

The analytic approach was informed by Klinkman's *Competing demands in psychosocial care* model, which provides a framework for accessing determinants of clinical diagnosis of depression, and depression treatment conditional on clinical diagnosis of depression (ie, depression care).^32^ The conceptual model and available evidence suggested that patients’ attributes largely determine who receives depression care.^32^, ^33^ Relevant patient attributes include (a) sociodemographic characteristics (ie, race,^34^ age,^35^ income,^21^ educational attainment,^36^ employment status,^21^ rural or urban residence,^36^ and marital status)^35^; (b) clinical characteristics (ie, severity of depression,^37^ and cancer stage at diagnosis)^36^; and (c) other individual‐level characteristics (ie, health insurance coverage 38, 39 Charlson comorbidity index,^40^ and number of annual visits to primary care clinics).^41^ After reviewing studies in other patient populations, we hypothesized that age, African‐American race, low education, rural residence, being unmarried, unemployment, and low annual income were negatively associated with depression care among prostate cancer survivors.^34^, ^37^, ^42^, ^43^

## 2 METHODS

### 2.1 Study population and procedure

Longitudinal data from a population‐based cohort of North Carolinian prostate cancer survivors who were enrolled from 2004 to 2007 in the *North Carolina‐Louisiana Prostate Cancer Project* (PCaP, N = 1031) was assessed. In brief, PCaP is a multidisciplinary study of social, individual, and tumor‐level causes of racial differences in prostate cancer aggressiveness. 44, 45 North Carolinian research subjects were incident prostate cancer cases diagnosed on or after July 1, 2004 and identified using records from the North Carolina Central Cancer Registry. Only African‐American and European‐American survivors were enrolled in equal proportions (sampling weight = 1:0.44).^45^ North Carolinian research subjects were contacted between September 2004 and December 2007 to obtain questionnaire data, biological specimens, and permission to obtain medical records. North Carolinian research subjects also had up to three annual follow‐up interviews on the *Health Care Access and Prostate Cancer Treatment in North Carolina Study* (HCaP‐NC, 2008‐2011 [N = 805]). Interview questionnaires were completed by regular mail or by phone interview for the first contact (between September 2008 and August 2009), second contact (September 2009 to August 2010) and third contact (September 2010 to August 2011). Data from 804 research subjects were analyzed. The University of North Carolina at Chapel Hill's Office of Human Research Ethics approved this study (Study # 17‐0183).

### 2.2 Measures

#### 2.2.1 Identifying self‐reported clinical diagnoses and treatment of depression

During enrollment, research subjects self‐reported prior clinical diagnosis of depression, and prior/ongoing depression treatment.^45^ During follow‐up, research subjects were asked the following questions: if they had ever been told that they had depression or anxiety by a health professional; if they were receiving antidepressants; and if they had received psychotherapy since the prior survey contact. Research subjects’ responses were used to create time–varying binary indicators of any self‐reported clinical diagnosis of depression and self‐reported depression treatment during the index survey wave.

#### 2.2.2 Identifying probable depression

Short Form 12 (SF‐12) is a patient reported outcome measure commonly used to assess physical and mental aspects of health‐related quality of life. SF‐12 is a 12‐item instrument, and responses are scored, weighed, and aggregated to yield physical and mental composite scores that are between 0 and 100 (average scores are 50, and higher scores indicate better health). Villagut and colleagues showed that SF‐12 mental composite scores (SF‐12 MCS) of 48.9 or less are 74% sensitive and 83% specific for identifying the occurrence of major or persistent depressive disorders within the prior 12 months.^46^ An SF‐12 MCS threshold score of 48.9 was used to create a time‐varying binary indicator of probable depression in the prior 12 months (*probable* because the threshold score is not diagnostic). This indicator was used as the denominator when estimating the average and annual rates of clinical diagnosis of depression among research subjects.

#### 2.2.3 Predictors

Key explanatory variables included age at enrollment, race, educational attainment (up to high school or beyond high school), rural or urban residence (using the 2010 US Census classification),^47^ current marital status (currently married, previously married or never married), current employment status (not employed [retired and unemployed] or employed) and current annual income (<$20 000, $20 000‐$40 000, $40 001‐$70 000, or >$70 000). Control covariates included time (in years) since prostate cancer diagnosis, a time‐invariant binary indicator of prostate cancer stage (T1 vs T2/T3),^48^ and time‐varying indicators of Charlson comorbidity index (0‐1 vs ≥2),^49^ health insurance status (insured vs uninsured), annual number of primary care visits (≤3 vs >3, [with three being the average number of annual visits to primary care clinics among similarly aged men in the general population during the study period]),^50^ and depression severity. The indicator of depression severity was created by applying SF‐12 MCS threshold scores suggested by other researchers (ie, <32.8 for moderate or severe depression, 32.8‐43 for mild depression, >43‐48.9 for *plausible* subthreshold depression, and >48.9 for no depression).^51^, ^52^

### 2.3 Statistical analyses

#### 2.3.1 Assessing predictors and rates of clinical diagnosis of depression

Generalized estimating equations (GEE) with a binomial family, logit link, and independent correlation were used to evaluate the indicator of self‐reported clinical diagnosis of depression as a function of indicated key explanatory variables and control covariates.^53^ The model predicted the average and annual rates of clinical diagnosis of depression among research subjects with probable depression. Survey sampling weights were applied and an alpha of 0.05 was used to determine statistical significance. Sensitivity analyses used alternative GEE correlation structures (ie, unstructured and exchangeable). Alwhaibi and colleagues have shown that frequent primary care visits are associated with a greater chance of clinical diagnosis of depression.^41^ Conversely, two studies have shown that patients undergoing depression treatment make frequent primary care visits.^54^, ^55^ Reverse causation between self‐reported clinical diagnosis of depression and annual primary care visits was reduced by lagging the indicator of annual primary care visits by one survey wave.

#### 2.3.2 Assessing predictors and rates of depression treatment conditional on clinical diagnosis of depression

A subsample of research subjects reported their first clinical diagnosis of depression during follow‐up. GEE with a binomial family, logit link, and independent correlation was used to evaluate the indicator of self‐reported depression treatment as a function of indicated key explanatory variables and control covariates in the subsample.^53^ The model predicted average rates of depression treatment using single observations from periods when research subjects reported their first clinical diagnosis. Survey sampling weights were applied and an alpha of 0.05 was used to determine statistical significance. Sensitivity analyses used alternative GEE correlation structures (ie, unstructured and exchangeable). Annual primary care visits were lagged by one survey wave to minimize bias from reverse causation.

#### 2.3.3 Dealing with missing data

400 PCaP research subjects were lost to follow‐up before the end of HCaP‐NC.^30^ *T‐ and Chi‐square tests* showed that research subjects lost to follow‐up were more likely to be African American, uninsured, smokers, and low income earners with higher prostate cancer stages.^56^ *Logit regression* showed that loss to follow‐up was random conditional on observed variables.^56^, ^57^ Survey response rates were about 95% (with respect to analytic variables) during each survey contact. Missing observations from survey nonresponse occurred at random and were addressed via multiple imputation (with 50 imputed datasets for explanatory variables only).^58^, ^59^, ^60^ Details of the imputation process (including specifications and diagnostics) are available on request.

## 3 RESULTS

### 3.1 Descriptive statistics

Most research subjects were middle‐aged/elderly, lived in urban areas, and were enrolled shortly after prostate cancer diagnosis (Table 1). Both European‐American and African‐American research subjects had similar distributions of health insurance coverage and cancer stage at diagnosis. However, African‐American research subjects had lower income and less education. Over 10% of research subjects reportedly received a clinical diagnosis of depression prior to enrollment, and an additional 12% reportedly received their first clinical diagnosis of depression during follow‐up. Similarly, about 7% of research subjects reportedly received depression treatment prior to enrollment, and an additional 9% reportedly initiated depression treatment during follow‐up. All research subjects who reportedly received depression treatment also reportedly received a clinical diagnosis of depression.

**Table 1:** Descriptive statistics at enrollment (N = 804)

Characteristics	Size (%)
Was diagnosed with depression prior to enrollment	
No	721 (90%)
Yes	83 (10%)
Race	
European American	450 (56%)
African American	354 (44%)
Marital status	
Currently married	628 (78%)
Previously married	133 (17%)
Never married	42 (5%)
Educational attainment	
More than high school	350 (44%)
High school or less than high school	453 (56%)
Residence	
Urban	609 (76%)
Rural	195 (24%)
Employment status	
Retired	366 (46%)
Employed	399 (50%)
Unemployed	36 (4%)
Annual income	
>$70 000	265 (35%)
$40 001‐$70 000	208 (27%)
$20 001‐$40 000	186 (24%)
≤$20 000	102 (13%)
Age at enrollment	
40‐49 years	39 (5%)
50‐59 years	254 (32%)
60‐69 years	341 (42%)
70‐79 years	170 (21%)
Indicators of depression severity	
No depression	575 (72%)
Plausible subthreshold depression	91 (11%)
Mild depression	86 (11%)
Moderate or severe depression	46 (6%)
Health insurance status	
Insured	423 (53%)
Uninsured	381 (47%)
Cancer stage at diagnosis	
T1(ref)	493 (62%)
T2/T3	306 (38%)
Charlson comorbidity index	
0‐1 (ref)	615 (77%)
≥2	187 (23%)
Visits to primary care clinics	
≤3 visits per year	428 (61%)
>3 visits per year	271 (39%)

### 3.2 Rates of self‐reported clinical diagnosis and treatment of depression

**Figure 1:**
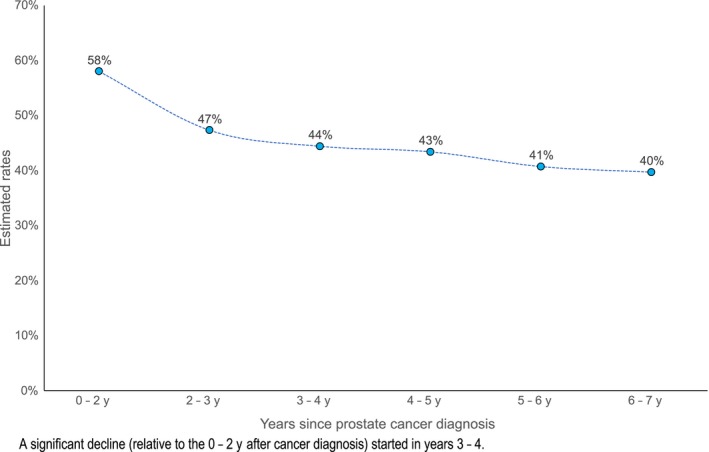
Estimated rates of self‐reported clinical diagnosis of depression during PCaP/HCaP‐NC

The average rate of self‐reported clinical diagnosis of depression among research subjects with probable depression was 44% (95% CI: 39%‐49%) over the study period (Table 2). The average rate of clinical diagnosis of depression was significantly lower among research subjects with the following characteristics: African American race; below college education; employed; and infrequent primary care visits. The rate of clinical diagnosis of depression among research subject with probable depression significantly declined from about 60% in the first 2 years after cancer diagnosis, to about 40% 3‐5 years later (Figure 1). The average rate of depression treatment conditional on clinical diagnosis of depression was 62% (95% CI: 55%‐69%) over the study period (Table 2). The average rate of depression treatment was similar across categories of race, educational attainment, employment status, and frequency of annual primary care visits.

**Table 2:** Estimated rates of self‐reported clinical diagnosis (conditional on probable depression) and treatment of depression (conditional on self‐reported clinical diagnosis of depression) among research subjects

Group	Outcomes			
	Clinical diagnosis of depression (N = 647 participant‐surveys)		Treatment of depression (N = 138 participant‐surveys)	
	Estimated rates	*P*‐Value	Estimated rates	*P*‐Value
All research subjects	44.3% (39.2‐49.4%)	—	62.0% (54.8‐69.2%)	—
By race				
European American	47.4% (41.2‐53.6%)	Ref	63.7% (55.0‐72.5%)	Ref
African American	37.7% (31.1‐44.4%)	0.02	56.1% (40.6‐71.6%)	0.43
By educational attainment				
≤High school	38.9% (32.0‐45.9%)	Ref.	60.2% (50.7‐69.7%)	Ref.
>High school	48.3% (41.9‐54.7%)	0.03	66.0% (49.6‐82.6%)	0.59
By employment status				
Retired/unemployed	48.6% (42.4‐54.9%)	Ref.	66.5% (56.4‐76.5%)	Ref.
Employed	40.0% (32.3‐45.7%)	0.02	56.3% (43.9‐68.7%)	0.24
By annual primary care visits				
At most three	39.0% (33.2‐44.8%)	Ref.	69.7% (58.0‐81.4%)	Ref.
Four or more	49.2% (43.3‐55.1%)	<0.01	55.0% (43.9‐66.2%)	0.10

Research subjects with probable depression had Short‐form 12 mental composite scores ≤48.9.^46^

Abbreviations: dep, depression; Ref, reference group.

### 3.3 Predictors of self‐reported clinical diagnosis and treatment of depression

Variables with significantly lower odds of self‐reported clinical diagnosis of depression included African‐American race; employment; age at enrollment; living with prostate cancer diagnosis for three or more years; having high school education or less; decreasing depression severity; and infrequent primary care visits. There was no evidence of significant associations between self‐reported clinical diagnosis of depression and any other variable in the model (Table 3). Variables with significantly lower odds of self‐reported depression treatment included decreasing depression severity, being employed and living with a prostate cancer diagnosis for more than 2 years. There was no evidence of any statistically significant association between self‐reported depression treatment and any other variable in the model.

**Table 3:** Factors associated with depression care among research subjects. Indicators of care were self‐reported clinical diagnosis of depression (N = 804 [or 2156 participant‐surveys]) and self‐reported depression treatment among research subjects with a self‐reported clinical diagnosis of depression during follow‐up (N = 132 [or 359 participant observations])

Outcomes	Depression care	
Variables	Diagnosis	Treatment
	*Odds ratios* (95% CI) *N = 804*	*Odds ratios* (95% CI) *N = 132*
Race		
European American (ref)	—	—
African American	0.63*	0.70
	(0.42‐0.94)	(0.35‐1.42)
Educational attainment		
High school or below	—	—
Above high school	1.56*	1.14
	(1.03‐2.35)	(0.54‐2.41)
Employment status		
Retired/unemployed (ref)	—	—
Employed/yet to retire	0.63*	0.49*
	(0.43‐0.93)	(0.25‐0.96)
Health insurance coverage		
Insured (ref)	—	—
Uninsured	0.57	2.07
	(0.22‐1.50)	(0.44‐9.70)
Cancer stage at diagnosis		
T1: a‐c (ref)	—	—
T2/T3: a‐c	0.82	0.64
	(0.56‐1.21)	(0.32‐1.26)
Age at enrollment		
	0.95**	0.99
	(0.93‐0.98)	(0.95‐1.04)
Time since cancer diagnosis		
13‐24 months (ref)	—	—
25‐36 months	0.61	0.14**
	(0.33‐1.11)	(0.04‐0.51)
37‐48 months	0.53*	0.22*
	(0.28‐0.98)	(0.06‐0.84)
49‐60 months	0.50*	0.22*
	(0.26‐0.98)	(0.05‐0.91)
61‐72 months	0.44*	0.17*
	(0.22‐0.88)	(0.04‐0.76)
73‐84 months	0.42	0.27
	(0.18‐1.01)	(0.04‐1.83)
Marital status		
Currently married (ref)	—	—
Previously/never married	1.23	1.12
	(0.78‐1.91)	(0.50‐2.51)
Residence		
Mostly urban (ref)	—	—
Mostly rural	1.24	1.42
	(0.80‐1.92)	(0.68‐2.93)
Charlson comorbidity index		
0‐1 (ref)	—	—
≥2	1.08	0.72
	(0.78‐1.49)	(0.42‐1.25)
Visits to primary care clinics		
≤3 visits per year (ref.)	—	—
>3 visits per year	1.62**	1.42
	(1.24‐2.12)	(0.82‐2.45)
Depression severity		
No depression (ref)	—	—
Plausible subthreshold dep.	2.10**	2.19
	(1.43‐3.09)	(0.97‐4.97)
Mild depression	5.08**	3.77**
	(3.42‐7.55)	(1.72‐8.22)
Moderate/severe depression	13.04**	4.93**
	(7.48‐22.73)	(2.22‐10.96)
Annual income		
>$70 000 (ref)	—	—
$40 001‐$70 000	0.80	1.05
	(0.52‐1.22)	(0.44‐2.50)
$20 001‐$40 000	0.84	0.65
	(0.50‐1.42)	(0.25‐1.67)
≤$20 000	0.93	0.46
	(0.50‐1.71)	(0.14‐1.53)

50 imputed datasets were used.

Abbreviations: CI, confidence interval; Dep, depression; ref, referent.

* *P*‐value ≤ 0.05.

** *P*‐value ≤ 0.01.

### 3.4 Sensitivity analyses

The findings were robust to GEE with exchangeable or unstructured correlations and to complete case analyses. Emerging evidence suggests reverse causation exists between depression severity and clinical diagnosis of depression: Simon and colleagues (1999) argue that depression severity predicts clinical diagnosis of depression^61^; Hung and colleagues (2017) showed that the duration of undiagnosed depression predicts depression severity.^62^ Study findings remained robust even after depression severity had been lagged by one survey wave to minimize bias from reverse causation.

## 4 DISCUSSION

### 4.1 Study implications

The data demonstrated the existence and nature of barriers to depression care among prostate cancer survivors. These findings have several important implications.

The rate of clinical diagnosis of depression in the general population [ie, 47% [95% CI: 42%‐53%]) is similar to our estimate (ie, 44% [95% CI: 39%‐49%]).^18^ Additionally, the rate of depression treatment in the general population (ie, 52% [95% CI: 42%‐62%]) is also similar to our estimate (ie, 62% [95% CI: 55%‐69%]).^63^ These suggest that despite an expected reluctance to seek depression care,^27^, ^28^, ^29^ prostate cancer survivors use depression care resources similarly to the general population. However, considering rates of depression treatment in the cancer literature (eg, 73% by Alwhaibi and colleagues [2017],^41^ and 76% by Findley and colleagues [2012],^36^) there seems to be a tendency toward more depression treatment among cancer survivors.

Evidence from this study demonstrated a significant racial difference in self‐reported clinical diagnosis of depression. This racial difference may arise from unequal access to depression care providers: this is consistent with evidence from the general population,^34^ from cancer survivors at the VA,^64^ and from research showing that race does not affect providers’ decision to make a clinical diagnosis of depression.^33^ Furthermore, no association was found between race and self‐reported depression treatment, which also is consistent with evidence from studies on survivors with other cancer types.^36^, ^41^ Hence, it is plausible that unequal access to depression treatment in the general population does not extend to prostate (or other) cancer survivors.^39^, ^65^ Additionally, the previously reported racial difference in risk of probable depression among research subjects is more likely due to unequal access to depression care providers than to depression treatment after clinical diagnosis of depression.^30^

The estimated rate of clinical diagnosis of depression among research subjects with probable depression declined from about 60% to 40% between prostate cancer diagnosis and 5‐7 years later. This suggests that lack of clinical recognition of depression (a type of diagnostic error)^31^ increased over time, and demonstrates a growing unmet need for depression care in prostate cancer survivors. Similarly, Table 3 showed that clinical recognition of depression decreased with advancing age at enrollment, thus depressed elderly survivors may be more likely to remain undiagnosed (as cancer care may gradually “crowd‐out” the clinical attention and quality of care that cancer survivors receive for comorbidities [including depression],^22^, ^23^, ^24^ and uncertainty about who [between primary and cancer care providers] should be responsible for meeting mental health care needs of cancer survivors may be increasing).^66^, ^67^ Other predisposing factors to undiagnosed depression include fewer primary care visits (where depression care is usually initiated),^66^, ^67^, ^68^ low education (due to higher mental health stigma than in those with more education),^69^, ^70^ and being employed (due to workplace stigma and potential income loss).^20^, ^21^, ^71^

The negative association between employment and self‐reported depression treatment deserves special consideration because of plausible policy implications. Luber and colleagues (2001) showed that adults receiving depression treatment are expected to have more frequent office visits for psychotherapy.^55^ Depressed employees undergoing psychotherapy for depression may require up to 20 weekly 1‐hour sessions at sites of care.^72^, ^73^ Leaving work for psychotherapy sessions creates a potential for income loss, assuming one only gets paid for hours worked.^71^ Paid Sick Leave laws and policies, as well as short‐ and long‐term disability insurance provide protection against income loss.^74^, ^75^ However, Paid Sick Leave laws and policies cover short‐term health needs (ie, between 24 and 72 hours per year),^74^ and may not protect sicker employees (with moderate/severe or treatment‐resistant depression) or those who need to travel long distances to get psychotherapy.^71^, ^74^ Similarly, short‐ and long‐term disability insurance covers 3‐4 in 10 workers and replaces 60%‐80% of one's income.^75^, ^76^ Hence, depressed employees with limited or no protection against income loss may have to choose one of two mutually exclusive options: to receive depression treatment and lose income; or to forego depression treatment for a chance at spontaneous remission with or without accompanying productivity and/or job loss (productivity loss being mostly borne by employers).^71^, ^77^, ^78^ Loss aversion under *Prospect theory* dictates that most people in this situation will choose the latter option,^79^ which may explain the negative association between employment and depression treatment. If this is the case, providing more protection against income loss may increase depression treatment rates.

### 4.2 Strengths and limitations

This study has two main strengths. Several clinically relevant factors (eg, depression severity, comorbidities, and cancer stage) and two major contributors to racial differences in access to mental health care (ie, geography in North Carolina and health insurance coverage)] were controlled for in the models.^80^ Application of sample weights makes these findings generalizable to prostate cancer survivors in North Carolina. This study also has several weaknesses. These findings from up to 13 years ago may not apply to present day prostate cancer survivors: for the Mental Health Parity and Addiction Equity Act (2008) and the Affordable Care Act (2010) were introduced after PCaP and reportedly increased access to depression care (ie, they would have led to higher rates of clinical diagnosis and treatment of depression among research subjects).^81^, ^82^, ^83^ What is unclear is if these Acts significantly reduced sociodemographic differences in access to depression care services for prostate (or all) cancer survivors. Self‐reported clinical recognition and treatment of depression is susceptible to error from misdiagnosis, recall bias and social desirability bias. Additionally, dependent variables in the GEE models may have been mislabeled in research subjects who reportedly received a clinical diagnosis of depression prior to enrollment and who had probable depression in an index wave^84^; and when assuming that self‐reported depression treatment was for depression rather than for anxiety disorders or chronic pain.^85^, ^86^ These errors may bias regression estimates towards the null or increase variance and risk of type 2 error. However, risks of bias and type 2 error are likely to be minimal for three key reasons. First, studies examining clinical diagnosis of depression using self‐reports and medical records demonstrated an 80% positive agreement (range: 51%‐100%) and 90% negative agreement (range: 71%‐100%).^87^, ^88^, ^89^ Second, the estimated rates of clinical diagnosis and treatment of depression in research subjects are consistent with the literature (described above).^18^, ^36^, ^41^, ^63^ Third, adults experiencing anxiety disorders or chronic pain are likely to be depressed.^2^, ^90^ Finally, the sample did not include prostate cancer survivors with late stage cancer at diagnosis—hence our findings do not extend to late stage disease.

## 5 CONCLUSION

Prostate cancer survivors experience access‐related barriers when in need of depression care. Factors associated with lower odds of clinical diagnosis of depression include African‐American race, being employed, older age at enrollment, low education, infrequent primary care visits and living with a prostate cancer diagnosis for three or more years. Factors associated with lower odds of depression treatment after receiving a clinical diagnosis of depression include being employed, decreasing depression severity, and living with a prostate cancer diagnosis for more than 2 years.

## CONFLICT OF INTEREST

None to declare.

## AUTHORS CONTRIBUTIONS

Daniel O. Erim was involved in conceptualization, investigation, formal analyses, writing—original draft, project management. Jeannette T. Bensen, James L. Mohler, Elizabeth TH Fontham, Lixin Song, Laura Farnan, Scott E. Delacroix, and Edward S. Peters were involved in investigation, writing—review and editing. Theodora N. Erim and Bradley N. Gaynes were involved in supervision, writing—review and editing. Ronald C. Chen was involve in investigation, supervision, writing—review and editing.

## Data Availability

The data that support the findings of this study are available from PCaP/HCaP‐NC management team. Restrictions apply to the availability of these data, which were used under license for this study.
